# P096 Mononeuritis multiplex in primary Sjögren’s syndrome: a diagnostic challenge in the presence of mechanical and functional pain disorders

**DOI:** 10.1093/rap/rkag113.126

**Published:** 2026-11-03

**Authors:** Ching Hua Chai, Tanjina Nasrin, Leslie Goh

**Affiliations:** Portsmouth Hospital University NHS Trust, Portsmouth, United Kingdom; Portsmouth Hospital University NHS Trust, Portsmouth, United Kingdom; Portsmouth Hospital University NHS Trust, Portsmouth, United Kingdom

## Abstract

**Introduction:**

Primary Sjögren’s syndrome (pSS) is a chronic systemic autoimmune inflammatory disorder characterised by lymphocytic infiltration of exocrine glands, most commonly presenting with sicca symptoms. However, the disease can manifest with a broad range of extraglandular features involving the skin, musculoskeletal, neurological, respiratory, gastrointestinal and renal systems. Neurological involvement is a recognised but relatively uncommon manifestation and can pose significant diagnostic challenges. We present the case of a 40-year-old woman with pSS who developed biopsy-proven mononeuritis multiplex in the context of longstanding mechanical back pain and fibromyalgia, highlighting the importance of recognising atypical neurological manifestations of Sjögren’s syndrome.

**Case description:**

A 40-year-old woman with primary Sjögren’s syndrome (pSS) was initially referred to Rheumatology in 2010 following ENT assessment for sicca symptoms. Salivary gland ultrasound demonstrated bilateral parotid sialectasis, while sialography showed multiple focal duct dilatations and strictures. Serological investigations revealed positive ANA (5.1), anti-Ro and anti-La antibodies, dsDNA of 30 IU/mL and rheumatoid factor >500 IU/mL. She reported dry eyes, dry mouth, fatigue, widespread myalgia and arthralgia. Hydroxychloroquine was commenced in 2011, resulting in significant improvement in sicca symptoms, fatigue and arthralgia. Over subsequent years, she developed intermittent paraesthesia affecting the right hand and chronic lower back pain. MRI of the lumbar spine demonstrated L5/S1 spondylolisthesis with foraminal narrowing and associated sciatica, requiring steroid facet joint injections. She was also diagnosed with fibromyalgia. In 2017, she developed bilateral hand and upper arm pain associated with burning sensations affecting both heels. Neurological examination demonstrated impaired coordination with reduced pin-prick and fine-touch sensation over the soles of both feet. Nerve conduction studies (NCS) demonstrated bilateral carpal tunnel syndrome and chronic denervation involving the right vastus medialis, tibialis anterior and medial gastrocnemius muscles, suggesting possible lumbosacral root involvement.By 2020, she experienced progressive neurological symptoms, including painful dysaesthesia affecting the left ulnar nerve distribution, intermittent inability to walk, sensory loss in the right foot, nocturnal burning pain and muscle cramping. Repeat NCS demonstrated absent superficial peroneal sensory responses, EMG shows axonal loss on right tibialis anterior, suggestive of mononeuritis multiplex. Given the progressive multifocal neuropathy, a sural nerve biopsy was performed and confirmed peripheral nerve vasculitis. She was treated with high-dose corticosteroids and cyclophosphamide, which was discontinued due to intolerance. Subsequent therapies included methotrexate, mycophenolate mofetil, rituximab and leflunomide. Her condition is currently clinically stable on leflunomide.

**Discussion:**

This case illustrates the diagnostic complexity of neurological involvement in primary Sjögren’s syndrome (pSS). Although the patient initially presented with classical manifestations of pSS, including sicca symptoms, fatigue, arthralgia and positive autoantibodies, the development of neurological symptoms evolved gradually over many years and was difficult to distinguish from several coexisting conditions.

The presence of established L5/S1 spondylolisthesis with foraminal narrowing, chronic mechanical back pain and sciatica provided a plausible explanation for many of her lower limb symptoms. In addition, fibromyalgia contributed to widespread pain and sensory complaints. The coexistence of vitamin B12, folate and vitamin D deficiencies, together with a history of probable illicit drug use, further broadened the differential diagnosis and complicated clinical assessment.

Despite these potential explanations, her symptoms continued to progress, with multifocal sensory deficits, severe neuropathic pain and functional impairment affecting different peripheral nerve distributions. Importantly, inflammatory markers were only mildly elevated during periods of neurological deterioration, with CRP ranging from 1–20 mg/L and ESR from 8–26 mm/hour. This made it challenging to determine whether her symptoms reflected active Sjögren’s disease.

Repeated neurological assessment and serial nerve conduction studies were instrumental in identifying an evolving neuropathy that could not be fully explained by mechanical, metabolic or degenerative causes. Given the persistent diagnostic uncertainty and high clinical suspicion for an inflammatory neuropathy, sural nerve biopsy was undertaken and ultimately confirmed peripheral nerve vasculitis causing mononeuritis multiplex.

This case demonstrates the importance of maintaining vigilance for neurological complications in patients with pSS, even when classical symptoms appear well controlled and alternative explanations exist. It also highlights the value of integrating clinical assessment, neurophysiological testing and tissue biopsy to achieve diagnostic certainty, guide immunosuppressive therapy and prevent irreversible neurological disability.

**Key learning points:**

• This case highlighted several important learning points regarding the diagnosis and management of severe extraglandular primary Sjögren’s syndrome (pSS).

• Firstly, neurological manifestations of pSS can develop despite apparently well-controlled sicca and systemic symptoms and may be difficult to recognise when alternative explanations exist. In this patient, mechanical spinal disease, fibromyalgia, vitamin deficiencies and a history of probable illicit drug use all contributed to diagnostic uncertainty. The case reinforced the importance of maintaining a high index of suspicion for neurological involvement when symptoms evolve or cannot be fully explained by existing diagnoses.

• Secondly, inflammatory markers may not accurately reflect disease activity in pSS. Throughout the course of her neurological deterioration, CRP and ESR remained normal or only mildly elevated. This emphasised the importance of careful longitudinal clinical assessment and demonstrated that significant extraglandular disease activity can occur in the absence of markedly abnormal blood tests.

• Thirdly, obtaining a definitive diagnosis may require specialist investigations and multidisciplinary input. Serial nerve conduction studies identified an evolving neuropathy, while sural nerve biopsy ultimately confirmed peripheral nerve vasculitis causing mononeuritis multiplex. This case reinforced the value of pursuing further investigations when clinical suspicion remains high despite competing diagnoses.

• Management of severe pSS remains challenging. Although corticosteroids were effective in treating acute vasculitic manifestations, long-term disease control was complicated by intolerance or inadequate response to multiple immunosuppressive therapies, including cyclophosphamide, methotrexate and mycophenolate mofetil. Recurrent infections, including COVID-19, chest infections and parotid infections requiring hospital admission, further complicated management and highlighted the need to balance disease control against treatment-related risks.

• Finally, this case demonstrated the importance of multidisciplinary collaboration between rheumatology, neurology, ENT specialists, physiotherapists and specialist nurses, as well as the importance of shared decision-making when navigating complex treatment choices.

